# Isolation and Characterization of a Novel Phage for Controlling Multidrug-Resistant *Klebsiella pneumoniae*

**DOI:** 10.3390/microorganisms8040542

**Published:** 2020-04-09

**Authors:** Qin Peng, Meng Fang, Xushan Liu, Chunling Zhang, Yue Liu, Yihui Yuan

**Affiliations:** 1 Ministry of Education Key Laboratory for Ecology of Tropical Islands, College of Life Sciences, Hainan Normal University, Haikou 571158, China; pengqin1019@hainnu.edu.cn (Q.P.); fangmeng361@163.com (M.F.); liuxushan1124@163.com (X.L.); 15983244852@163.com (C.Z.); liuyue423520@163.com (Y.L.); 2 State Key Laboratory of Marine Resource Utilization in South China Sea, Hainan University, Haikou 570228, China

**Keywords:** phage, *Klebsiella pneumoniae*, multidrug-resistance, phage therapy

## Abstract

The emergence of multidrug-resistant bacterial pathogens has severely threatened global health. A phage with the ability to efficiently and specifically lyse bacteria is considered an alternative for controlling multidrug-resistant bacterial pathogens. The discovery of novel agents for controlling the infections caused by *K. pneumoniae* is urgent due to the broad multidrug-resistance of *K. pneumoniae*. Only a few phage isolates have been reported to infect multidrug-resistant *K. pneumoniae*. In this study, by using the multidrug-resistant *K. pneumoniae* strain as an indicator, a novel phage called vB_KleS-HSE3, which maintains high antibacterial activity and high physical stability, was isolated from hospital sewage. This phage infected one of four tested multidrug-resistant *K. pneumoniae* strains. This phage belongs to the Siphoviridae family and a comparative genomic analysis showed that this phage is part of a novel phage lineage among the Siphoviridae family of phages that infect strains of *Klebsiella*. Based on its features, the vB_KleS-HSE3 phage has potential for controlling infections caused by multidrug-resistant *K. pneumoniae.*

## 1. Introduction

*Klebsiella pneumoniae* is a ubiquitous Gram-negative nonmotile pathogen that belongs to the Enterobacteriaceae family. The strains of this species are widely distributed in the natural environment, including sewage, soil, plant surfaces, and the surface of medical devices [1,2]. Moreover, the strains of this species commonly show the ability to colonize the mucosal surfaces of humans, including the nasopharynx and the gastrointestinal (GI) tract [3,4], and thus are reported to cause a wide range of infections, including pneumonias, urinary tract infections (UTIs), bloodstream infections, and liver abscesses [5,6,7]. *K. pneumoniae*, together with *Enterococcus faecium*, *Staphylococcus aureus*, *Acinetobacter baumannii*, *Pseudomonas aeruginosa*, and *Enterobacter* species, is classified as an ESKAPE pathogen [8], which are considered one of the greatest challenges to global health due to their resistance to almost all available antibiotics [9,10].

The broad antibiotic-resistance of *K. pneumoniae* is attributed to their large accessory genome, which exists as plasmid and chromosomal gene loci in the strain and contains various antibiotic-resistant genes [11]. The high frequency horizontal gene transfers (HGTs) of the antibiotic-resistant genes, aided by plasmids and mobile genetic elements, further increase the multidrug-resistance of *K. pneumoniae* [12]. Two major types of antibiotic-resistant mechanisms are widely distributed among *K. pneumoniae.* The first one is the expression of extended spectrum β-lactamase (ESBL), which gives the bacteria resistance to cephalosporins and monobactams. The other, which is even more troubling, is the expression of carbapenemase, which renders the bacteria resistant to almost all available β-lactams that are routinely used to cure hospitalized or immunocompromised patients against *Enterobacteriaceae* pathogens [3]. Furthermore, the pathogen’s multidrug resistance to newly developed antibiotics makes the treatment of *K. pneumoniae* infections much more difficult [13]. Over the last few decades, polymyxin E (colistin) has been considered as a “last resort” antimicrobial agent to fight multidrug-resistant *K. pneumoniae* [14]. However, recent reports on the resistance of *K. pneumoniae* to colistin have further limited the strategies for controlling infections caused by *K. pneumoniae*, leading to the high mortality rates of such infections [14].

With the ability to lyse bacterial pathogens within a short lysis time, phage and phage encoded endolysin have been reconsidered as alternatives for treating the infections caused by tenacious pathogens [15,16,17,18,19]. Compared with antibiotics, phage therapy exhibits numerous advantages, including a highly specific lysis spectrum, co-evolution with bacteria to avoid the emergence of phage-resistance, and a higher abundance and diversity of phage resources than found in antibiotics [20,21]. Meanwhile, the construction of a phage cocktail by using phage strains with different genetic backgrounds could further limit the appearance of phage resistance during practical application [22,23,24,25]. Attesting to the advantages of phage therapy, several bacterial infections, including the infections caused by multidrug-resistant pathogens that cannot be treated by antibiotics, have been cured by phages [26]. Due to its high antibiotic-resistance, numerous phages have been isolated to control the infections caused by *K. pneumoniae* [27,28,29,30,31]. Until January 16, 2020, according to the records of phage genomes at the National Center for Biotechnology Information (NCBI), 80 phage strains of the Siphoviridae family that can infect the *Klebsiella* genus strains have been isolated [29,30,31,32]. However, due to the high genomic similarity between phage strains, the currently available *K. pneumoniae* phages have low genetic diversity. Thus, the isolation of a *K. pneumoniae* phage with low genomic similarity to other available phages is very important.

In this study, a novel phage called vB_KleS-HSE3, which can infect the multidrug-resistant *K. pneumoniae* strain, was isolated. This phage showed high efficiency in infecting multidrug-resistant host bacterium, as well as high physical stability. A comparative genomic analysis revealed that this phage represented a novel cluster of phages. Taking into considering all of the above features, the newly isolated vB_KleS-HSE3 phage is a promising alternative for controlling the infections caused by multidrug-resistant *K. pneumoniae.*

## 2. Materials and Methods

### 2.1. Phage Isolation and Propagation 

*K. pneumoniae* strains 1025, 2106, 0915, and 2404 were isolated from sputum samples of patients in Liyuan Hospital (Wuhan, Hubei, China), and antimicrobial susceptibility tests of these strains were conducted by using the Kirby–Bauer disk diffusion method according the Clinical and Laboratory Standards Institute (CLSI) guidelines 2018 [33]. *K. pneumoniae* strain 1025 was used as the indicator strain for isolation of the phage [34]. 

Sewage samples collected from Liyuan Hospital were used to isolate the phage that infected *K. pneumoniae* strain 1025. The bacterial strain used in this study was grown in a Luria–Bertani (LB) broth medium at 37 °C. The sewage samples were first centrifuged at 12,000× *g* for 10 min to remove their solid impurities, and then the supernatants were filtered through a 0.22 μm pore-size membrane filter to remove the bacterial debris. The filtered supernatants were further added into the cultures of the exponential growth *K. pneumoniae* strain 1025 and co-cultivated for an additional 8 h. After cultivation, the cultures were centrifuged at 8000× *g* at 4 °C for 30 min, and the supernatants were used for phage isolation, as previously described, via the double-agar overlay method after filtering through a 0.22 μm pore-size membrane filter [35]. The 100 μL filtrate and 200 μL exponential growth bacterial culture was mixed for 5 min and then added into 4 mL of a melted semisolid LB medium. The mixture was further overlaid onto an LB agar plate, and the phage plaque was chosen. The chosen phage plaque was further purified by using the double-ager overlay method at last 5 times until the formation of uniform phage plaques. The efficiency-of-plating was also tested by using the double-agar overlay method [36]. 

### 2.2. Electron Microscopy Observation of Phage Virions

To observe the virion morphology of the phage, the phage plaques formed on the agar plate were washed off by using an SM buffer (10 mM Tris, 100 mM NaCl, and 10 mM MgSO_4_, pH 7.5) and further filtered through a 0.22 μm pore-size membrane filter for use. After negative staining with 2% potassium phosphotungstate (pH 7.2), the phage morphology was imaged by transmission electron microscopy (TEM, G20, Tecnai, FEI, Hillsboro, OR, USA) at an acceleration voltage of 200 kV [36].

### 2.3. Host Range Determination

To determine the host range of the phage isolated in this study, strains belonging to the species of *K. pneumoniae*, *Escherichia coli*, *Acinetobacter baumannii*, *Staphylococcus aureus*, and *Yersinia pseudotuberculosis* were used. The host range was determined in a single experiment with technical triplicates by using the previously described method [37]. For each strain, the exponential growth cultures were mixed with a melted semi-solid medium and overlaid onto an LB agar plate. The suspension of the purified phage with a concentration of 10^8^ PFU/mL was added onto the upper semisolid medium at a volume of 1 μL, and the formation of phage plaques was observed after being cultivated at 37 °C overnight. 

### 2.4. One-Step Growth Curve

To analyze the one-step growth curve of the phage isolated in this study, the phage suspension was added into the culture of the exponential growth host strain at a multiplicity of infection (MOI) of 1.0 and further mixed at 37 °C for 5 min for the adsorption of the phage to the bacterial cell. Subsequently, the mixture was centrifuged at 10,000× *g* for 1 min to remove the non-absorbed phage. Then, the pellet was resuspended in 50 mL fresh LB broth, and the phage titers in the culture were determined at an interval of 15 min. The burst size of the phage was determined as previously described in a single experiment with technical triplicates [34]. 

### 2.5. Physical Stability of the Phage

The temperature stability of the isolated phage was determined at 4 °C, 28 °C (representing room temperature for phage storage), 37 °C, 50 °C, 60 °C, and 70 °C, respectively, by placing the newly prepared phage suspension at each temperature for 30 min. The newly prepared phage suspension was stored at 4 °C, and the phage concentration was 10^13^ PFU/mL before treatment. After the treatment, the phage suspension was cooled slowly, and the titers of the phage in each treated suspension were determined by the double-agar overlay method. The pH tolerance of the isolated phage was tested by incubating the phage in buffers with a pH of 1.0, 3.0, 5.0, 7.0, 9.0, and 11.0 for 30 min at 28 °C. The results were expressed as a percentage of the initial viral counts. For each assay, the phage was treated by different treatments once, the phage titer was tested three times, and the means were used.

### 2.6. Phage DNA Isolation and Genome Sequencing

The genomic DNA of the isolated phage was extracted as previously described via phenol–chloroform extraction with protease K-sodium dodecyl sulfate (SDS) treatment [38]. The purified phage genomic DNA was sequenced by using an Illumina HiSeq 2500 sequencer. A total of 9,293,738 reads were assembled into contigs by use the SPAdes-3.5.0 software (Illumina, San Diego, CA, USA). Gaps between contigs were filled by prime walking. The coding sequences (CDSs) in the genome were predicted using the FGENE SV0 software (Softberry, http://linux1.softberry.com) and by visual verification. Each predicted gene was annotated by performing a search in the NCBI non-redundant protein sequences (NR) and CDD databases using the basic local alignment search tool (BLAST) [39], combined with an analysis of the motif and functional domain composition of the predicted protein with the Pfam and HHpred database [40]. Phage genome annotation was visualized by using Easyfig 2.2.2 [41]. The genes encoding putative tRNAs were searched using tRNAscan-SE 2.0 (http://lowelab.ucsc.edu/tRNAscan-SE/) [42]. A phylogenetic analysis of the proteins was performed using MEGA X with the neighbor-joining method and a bootstrap analysis (1000 replicates) with the ClustalW alignment [43], visualized using Fig Tree v1.4.4. Visualization of the phage comparative genome was performed using Circoletto (http://tools.bat.infspire.org/circoletto/) [44], and a comparative genomic analysis of the phage genome was performed using Gepard-1.40 [45]. The CoreGenes 3.5 (http://binf.gmu.edu:8080/CoreGenes3.5/custdata.html) was used to analyze the core genes of the phages, and genes with scores higher than 75 were considered to be core genes [46]. The annotated genome sequence of vB_KleS-HSE3 phage was deposited in GenBank under accession number MT075871.

## 3. Results

### 3.1. Microscopic Morphology of the Phage Virion

**Figure 1:**
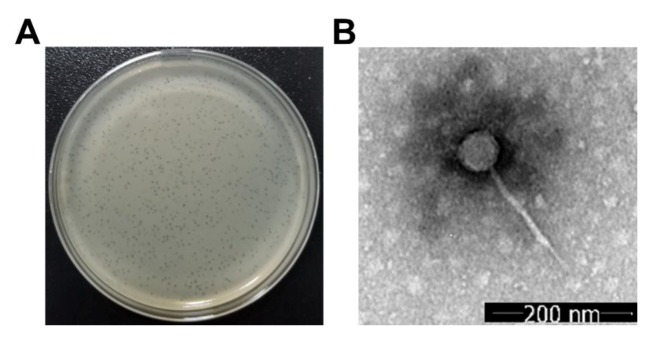
Morphological observation of the vB_KleS-HSE3 phage. (**A**) The plaques formed by the phage on the bacterial lawn of *K. pneumoniae* strain 1025. (**B**) Virion morphology of the phage observed by TEM.

By using the sewage sample collected from the hospital, one phage that formed a transparent round plaque with a diameter of about 0.5 mm on the bacterial lawn of *K. pneumoniae* strain 1025 was isolated (Figure 1A). This phage was named vB_KleS-HSE3. The microscopic observation of the virion morphology by TEM showed that the vB_KleS-HSE3 phage harbors an icosahedral head 54 nm in diameter with a tail 189 nm long and 8 nm wide (Figure 1B). Based on the microscopic morphology of the phage virion, this phage was classified as part of the Siphoviridae family.

### 3.2. Host Range of vB_KleS-HSE3 Phage

The host range analysis of the vB_KleS-HSE3 phage showed that the phage could only infect the tested *K. pneumoniae* strain 1025 and one strain of the *Y. pseudotuberculosis* species but not the other tested *K. pneumoniae* strains or strains belonging to other species (Table 1). Interestingly, this phage could also infect the strain of the *Y. pseudotuberculosis* species, which also belongs to the Enterobacteriaceae family and might contain receptors for the infection of the phage. The four *K. pneumoniae* strains used in this study were all isolated from the sputum samples of patients in the hospital. However, they maintained different antibiotic-resistance levels. Strain 1025 showed the broadest resistance to the tested antibiotics, including β-lactam antibiotics (aztreonam, piperacillin, piperacillin-tazobactam, cefazolin, cefuroxime, cefotaxime, ceftriaxone, cefepime, cefoxitin, cefoperazone-sulbactam, and ampicillin-sulbactam), quinolones antibiotics (ciprofloxacin, levofloxacin, and norfloxacin), carbapenems antibiotics (imipenem and meropenem), and aminoglycosides antibiotics (amikacin, gentamicin, and tobramycin) (Table 2). 

### 3.3. One-Step Growth Curve

**Figure 2:**
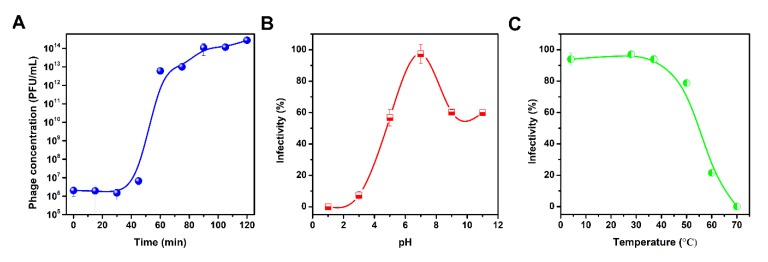
Characterization of the vB_KleS-HSE3 phage. (**A**) One-step growth curve of the phage. (**B**) Tolerance of the phage to different pH treatments. (**C**) Thermal tolerance of the phage. For each test, the titer of the phage was tested by three replicates, and the mean value was used. The standard deviation of each datum is shown.

The one-step growth curve analysis of the vB_KleS-HSE3 phage revealed that the vB_KleS-HSE3 phage maintained a latent period of approximately 30 min and that the multiplication period reached a plateau at 90 min, indicating the fast lysis speed of the phage (Figure 2A). At 90 min, the phage concentration reached up to 10^14^ PFU/mL. The calculated burst size of the vB_KleS-HSE3 phage was about 277 PFU per bacterial cell based on the phage concentration at 90 min. This result indicates that the phage maintained a high replication capacity, and could produce more progeny phage to control the infections caused by *K. pneumoniae*. This high reproductive ability would benefit the practical application of this phage.

### 3.4. Thermal and pH Tolerance of vB_KleS-HSE3 

Except for the lysis performance of the phage to the bacterial pathogen, the physical tolerance of the phage was also critical for the application of the phage in different conditions. The thermal and pH tolerance analyses of vB_KleS-HSE3 showed that the phage maintained high tolerance against a broad pH and thermal range. In a pH range between 5.0 and 11.0, more than 55% of the phage remained viable after being treated for 30 min (Figure 2B). A sharp decrease in phage viability was observed at pH 3.0, possibly because the low pH caused denaturation of the phage’s virion proteins. The phage also maintained high viability below the temperature of 50 °C, and less than 20% of the phage lost viability after being treated for 30 min (Figure 2C). At a temperature of 60 °C, most of the phage lost viability, and the phage viability was totally lost at 70 °C. The high thermal and pH tolerance of the phage broadens the practical application condition of the phage.

### 3.5. Genome Analysis of vB_KleS-HSE3 Phage 

The vB_KleS-HSE3 phage has a double-stranded linear DNA genome 46,747 bp in length, and the G+C content of the phage genome is 56.47%. In total, the gene annotation of the phage genome showed that the genome of the vB_KleS-HSE3 phage contains 67 open reading frames (ORFs) and no tRNA (Table S1). Among these 67 genes, 39 were transcribed in a forward direction, while the other genes were transcribed in reverse. A comprehensive search of the NR database for homologs of the 67 ORFs returned 51 significant matches (E-value ≤ 10^−3^), including 45 that were similar to genes of the *K. pneumoniae* phage vB_Kp3, and 16 that were specific to the vB_KleS-HSE3 phage. The putative functions of the phage proteins were predicted by a bioinformatic analysis, which showed that 27 coding domain sequences (CDSs) were functionally annotated, while 40 of the 67 CDSs were annotated as hypothetical proteins. The length of the CDSs encoded by the vB_KleS-HSE3 phage ranged from 68 to 437 amino acid residues, with an average length of 247.63 residues. 

### 3.6. Modular Organization of the vB_KleS-HSE3 Phage Genome 

**Figure 3:**
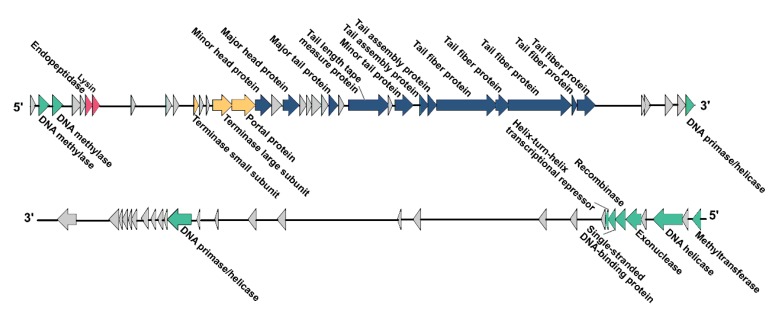
Genome organization of the vB_KleS-HSE3 phage. Different colors indicate four different gene modules: the DNA packaging gene module (orange color), the structural gene module (dark blue color), the nucleic acid metabolism associated gene module (light green color), and the cell lysis associated gene module (red color). Genes with unknown functions are indicated by a gray color.

The functional analysis of the phage ORFs showed that the genes of the phage form a modular structure, including the modules that encode the structural gene cluster (12 ORFs), the DNA packaging associated gene cluster (3 ORFs), the nucleic acid metabolism associated gene cluster (10 ORFs), and the host cell lysis associated gene cluster (2 ORFs) (Figure 3). Genes with associated functions were located close together in the genome, which might benefit the regulation of the phage’s life cycle after infecting the host bacterium. Except for the genes associated with the metabolism of nucleic acids, the other three gene modules were transcribed in the same direction and in the same DNA strand. The different transcription directions and locations of the genes associated with the metabolism of nucleic acids might be due to the different expression times of these genes after infection.

#### 3.6.1. Functions of Genes in Structural Gene Module

**Figure 4:**
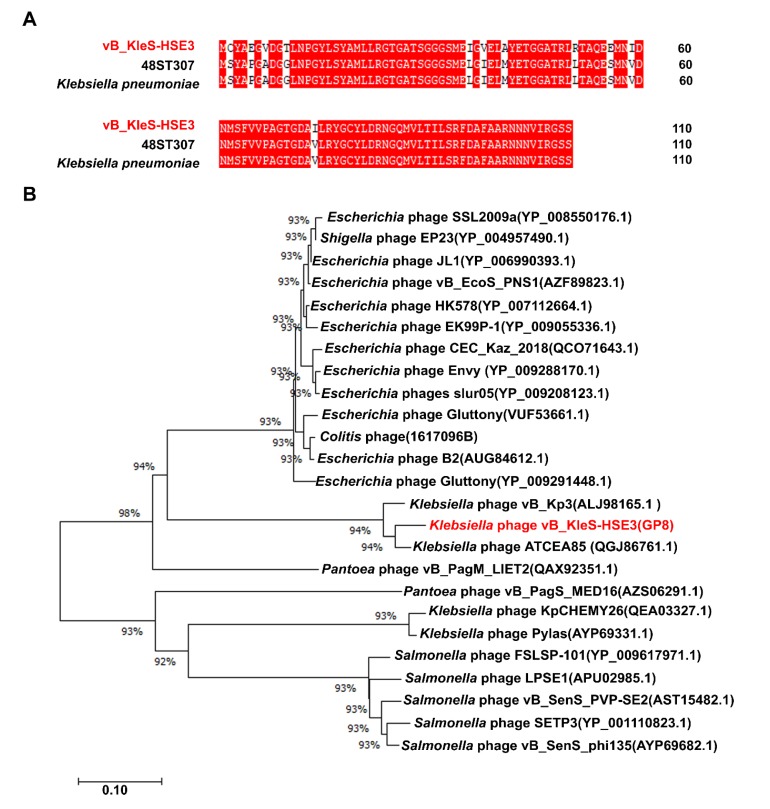
Analysis of the functional proteins Gp52 and Gp8 of the vB_KleS-HSE3 phage. (**A**) Alignment of the protein Gp52 with the proteins of the *Klebsiella* phage 48ST307 (Genbank Accession No. AQN32348.1) and the *K. pneumoniae* bacterial strain (Genbank Accession No. WP_135718102.1). (**B**) Phylogenetic analysis of the lysins that showed a high similarity to the lysin of the vB_KleS-HSE3 phage. The phylogenetic tree was generated by using the neighbor-joining method and bootstrap analysis (1000 replicates) in MEGA X. Only lysins that showed more than a 40% similarity with Gp8 were used. The scale bar represents 0.10 substitutions per nucleotide position.

Among the 27 genes with putative functions, 12 were annotated as structural protein encoding genes, including the genes *gp*30, *gp*33, *gp*38, *gp*40, *gp*43, *gp*45, *gp*46, *gp*47, *gp*48, *gp*49, *gp*52, and *gp*53 (Figure 3). The functions of these 12 gene were essential for the synthesis of the phage particle, including the head (2 ORFs), tail (5 ORFs), and tail fiber (5 ORFs). The gene product of *gp*30 was a minor head protein, which was required for viral head morphogenesis in the phage [49]. The similarity analysis showed that 11 of these 12 genes were consistent with the distribution and sequence of the structural module genes of the *Klebsiella* phage vB_Kp3, while the gene *gp*52, which encodes the phage tail fiber protein, showed a higher similarity (89.19% similarity) to the gene of the *Klebsiella* phage 48ST307 and the genes of the host *K. pneumoniae* bacterial strain (Figure 4A).

#### 3.6.2. Functions of Genes in the Genome Packing Associated Gene Module

Three genes in the genome of vB_KleS-HSE3 phage were annotated as phage genome packing associated genes, including *gp*27, *gp*28, and *gp*29, which are all located upstream from the structural gene module. The phage DNA packaging process mainly relies on complex machinery consisting of terminase and portal proteins. The portal protein is located at the vertex of the phage empty head by forming a portal channel for the entrance of the phage genome DNA, and the terminase is docked on the portal to recognize and deliver the phage genome’s DNA [50]. 

#### 3.6.3. Functions of Genes in the Cell Lysin Associated Gene Module

Two genes were annotated as cell lysis associated genes, *gp*7 and *gp*8, which were predicted to encode endopeptidase and lysin, respectively. The function of these two gene products is to degrade the bacterial cell wall for the release of mature phage virions [51]. The phage host lysis gene module usually contains additional holin protein, whose function is to form pores on the bacterial cell membrane for the bypass of lysin [36]. However, no holin gene was identified in the genome of the vB_KleS-HSE3 phage, suggesting that the lysin of the vB_KleS-HSE3 phage passes through the bacterial cell membrane via another strategy. The host lysis gene module of the vB_KleS-HSE3 phage is highly similar to that of the *Klebsiella* phage ATCEA85 and the *Klebsiella* phage vB_Kp3, but not the other *Klebsiella* phage. The lysin Gp8 showed a high similarity to the lysins of the *Klebsiella* phage ATCEA85 (94.55%, GenBank No. QGJ86761.1) and the *Klebsiella* phage vB_Kp3 (92.73%, GenBank No. ALJ98165.1), whereas similarities lower than 61% were observed for other phages, including the phages of *Klebsiella*, *Escherichia*, *Salmonella*, *Colitis*, and *Pantoea*. A phylogenetic analysis of the most similar lysins with the lysin encoded by the vB_KleS-HSE3 phage also confirmed the close relationship of the lysins in these three phages in evolution, which might be classified as a novel cluster of phage lysins (Figure 4B). 

#### 3.6.4. Functions of Genes in the Nucleic Acid Metabolism Associated Gene Module

The genomic annotation of the phage revealed that ten of the phage genes were associated with nucleic acid metabolism, including *gp*2, *gp*4, *gp*19, *gp*55, *gp*56, *gp*57, *gp*58, *gp*62, *gp*66, and *gp*67, which are mainly located at the termini of the phage genome. Unlike the genes of the other functional modules, seven of the ten genes were distributed on the other DNA strand, suggesting that two of the DNA strands maintained different functional divisions. One of the DNA strands of the vB_KleS-HSE3 phage only participated in the function of phage DNA metabolism, which ensures the successful synthesis of genomic DNA for progeny phages. Except for gene *gp*67, which was located at the end of the phage genome and showed a high similarity to the genes of the *K. pneumoniae* bacterial strain, the other genes were similar to the genes of the *Klebsiella* phage vB_Kp3. Gp67 was annotated as methyltransferase by searching against the NR database in NCBI. Considering of the location and the sequence similarity of gene *gp*67, it is rational to speculate that the gene was obtained from the host bacterium via genetic recombination during the replication of the phage genome in the bacterial cell.

### 3.7. Comparative Genomic Analysis of the vB_KleS-HSE3 Phage

**Figure 5:**
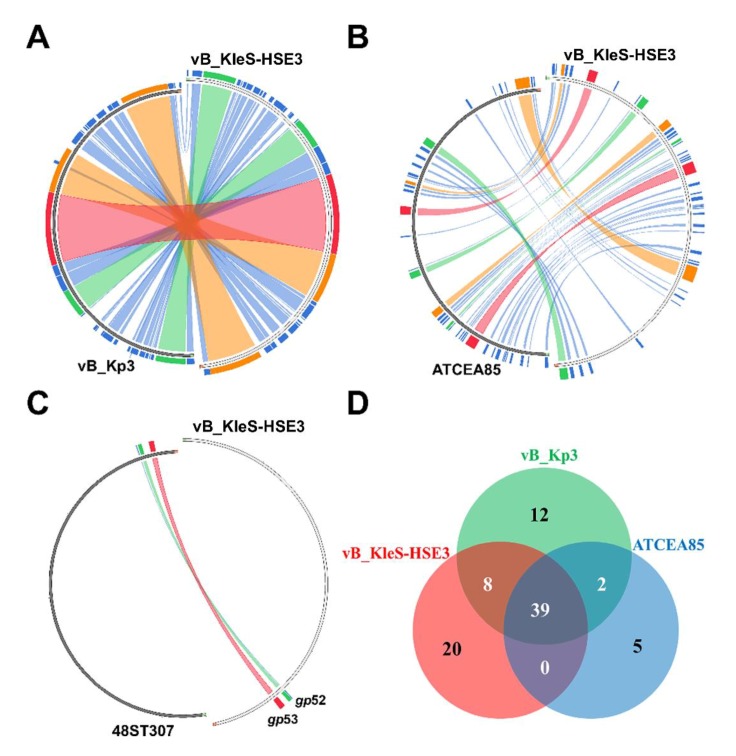
Comparative genomic analysis of the *Klebsiella* vB_KleS-HSE3 phage. The genome sequence of the *Klebsiella* vB_KleS-HSE3 phage was compared with that of the *Klebsiella* phage vB_Kp3 (**A**), ATCEA85 (**B**), and 48ST307 (**C**). The results were visualized using Circoletto, and the similar genome regions are shown in the same color. (**D**) The core gene analysis of *Klebsiella* phage vB_KleS-HSE3, vB_Kp3, and ATCEA85.

For a comparative genomic analysis of the vB_KleS-HSE3 phage, the genome of the phage was searched against the phage genome sequences in GenBank by using BLSATN in NCBI, and the result showed that the phage only maintains a high similarity to the *Klebsiella* phage vB_Kp3 (75.6% similarity) and the *Klebsiella* phage ATCEA85 (48.47% similarity) (Figure 5A,B). For the other phages, a genome similarity less than 3% was observed (Figure 5C). The core genes of the four *Klebsiella* phages, vB_KleS-HSE3, vB_Kp3, ATCEA85, and 48ST307, were analyzed, and the result revealed that five genes were conserved in these phages (Table S2). The gene products of these five genes were all structural proteins, including three tail proteins and two tail fiber proteins. For the three phages that exhibited a high similarity, including phage vB_KleS-HSE3, vB_Kp3, and ATCEA85, 39 genes were found to be conserved in their genomes (Table S3). Among them, 17 were predicted to encode hypothetical proteins and 22 were predicted to encode functional proteins, including nine structural proteins, eight nucleic acid metabolism associated proteins, three DNA packaging associated proteins, and two host cell lysis associated proteins. In total, twenty, twelve, and five unique genes were found in the genomes of phage vB_KleS-HSE3, vB_Kp3, and ATCEA85, respectively (Figure 5D). The unique genes in these three phage genomes mainly encoded proteins with unknown functions, except for the two genes of the vB_KleS-HSE3 phage, including *gp*52 and *gp*67, which were predicted to encode a phage tail fiber protein and methyltransferase. These two genes were found to be similar to the genes of the bacterial strain belonging to *K. pneumoniae* but not to the genes of the phage. Phage tail fiber protein was reported to responsible for the determination of the host specificity of the phage [52]. The differences in the phage tail fiber proteins of these phages lead to the different host ranges of these phages. Methyltransferase is a component of the bacterial restriction–modification (R–M) system, which catalyzes the methylation of the bacterial genome and protects the bacterial cell from the invasion of foreign DNA [53]. The phage-encoding methyltransferase catalyzes the methylation of the phage genome and protects the phage genome from being recognized and damaged by the bacterial R–M system, which would further benefit phage therapy by prolonging the effectiveness and broadening the host range.

**Figure 6:**
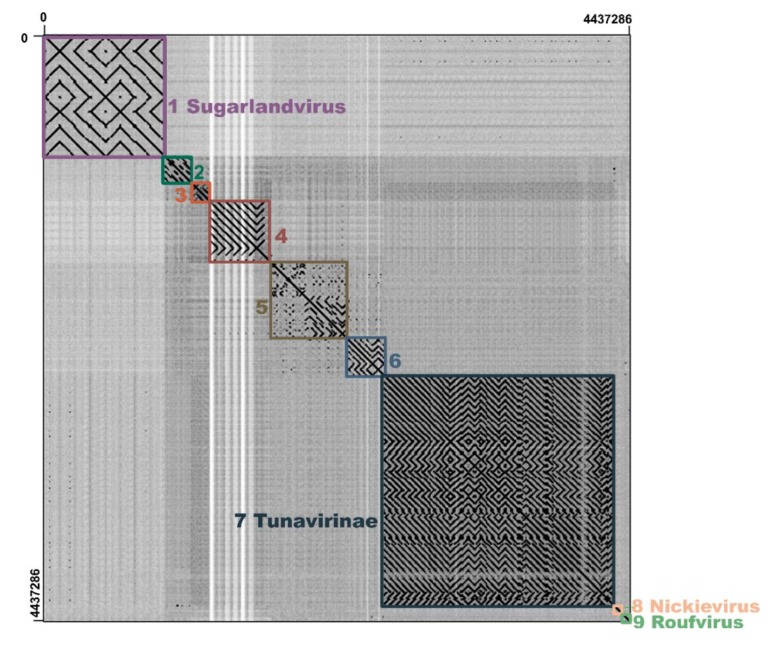
Dot plotting analysis of the genomes of the Siphoviridae family phages infecting *Klebsiella.* The phage genomes were used as the list in Table S4. The dot plotting analysis was performed by using Gepard.

To further analyze the genome similarities of the phages that infect *Klebsiella* genus bacterial strains, the genomes of all 80 *Klebsiella* phages belonging to the Siphoviridae family were compared by a dot plotting analysis (Figure 6). According to the results, 80 *Klebsiella* phages were classified into nine clusters; the phage vB_KleS-HSE3 together with the *Klebsiella* phage vB_Kp3 and ATCEA85 were grouped together as a novel phage cluster. The phages belonging to the cluster of the vB_KleS-HSE3 phage rarely exhibited similarities to the phages of the other groups, suggesting that the phages of this cluster represent a novel phage lineage.

## 4. Discussion

In this study, a novel phage vB_KleS-HSE3 was isolated. This phage showed high lytic activity to the multidrug-resistant *K. pneumoniae* strain 1025, which is resistant to almost all available antibiotics. The indicator strain used in this study was isolated from the sputum sample of patients in the hospital. Meanwhile, the phage was isolated from the sewage from the same hospital. The hospital sewage had a high possibility to contain both the phage and the host bacterium, allowing the phage to experience co-evolution with the host bacterium [35]. This type of co-evolution process was hypothesized to endow the phage with numerous mutations to infect bacterial pathogens of different phenotypes [23], and the phage mutants with different activities were thought to have been enriched in the hospital sewage. The phage isolated in this study only targets specific strains of the bacterial pathogen, but not all the strains of the same bacterial species isolated from the same hospital. Several other phages were also isolated from hospital sewage by using pathogenic bacteria from the same hospital as indicators [34,47]. The findings of this study indicate that the hospital sewage provides a resource for isolating phages that can target the multidrug-resistant bacterial pathogens.

Due to co-evolution of the phages and their host bacteria, the construction of a phage cocktail was able to avoid bacterial phage resistance [54]. Furthermore, the construction of a phage cocktail was also able to control the multiple infections caused by different bacterial pathogens [55]. To develop a phage cocktail, the isolation of phages with diverse genetic background was very important. However, so far, the Siphoviridae family phages that infect strains of *K. pneumoniae* have shown a highly conserved genetic background. The phage vB_KleS-HSE3 isolated in this study showed only a low similarity to phage vB_Kp3 and ATCEA853, which were grouped together as a novel cluster with the Siphoviridae family phage of *Klebsiella* spp., and similarities with other phages were rarely observed. The low genomic similarity of the phage isolated in this study with other phages suggests that the phage vB_KleS-HSE3 is able to be used in the construction of phage cocktails for medical application.

Although the phages vB_KleS-HSE3, vB_Kp3, and ATCEA853 contain similar genome sequences, the geographical distribution analysis revealed that these three phages were isolated from China, Switzerland, and Korea, respectively. In addition to these three phages, the other *Klebsiella* genus phages with high genomic similarities also showed a broad geographical distribution. For example, the eight phages classified as cluster one in this study were isolated from USA, Israel, Australia, and Russia (Table S4). According to a previous report, due to the overestimation of the differences between phages with different geographical distributions and the influence of geographical isolation in blocking phage diffusion, the diversity of phages has been highly overestimated [56]. This finding suggests that geographical isolation might only exert minimal influence on the global distribution of the phages.

## Figures and Tables

**Table 1:** Host range of the vB_KleS-HSE3 phage.

Strains	Phage Sensitivity ^a^	Strain Resource
*Klebsiella pneumoniae*		
1025	S	A clinical isolate [47]
2404	R	A clinical isolate
0915	R	A clinical isolate
2106	R	A clinical isolate
*Yersinia pseudotuberculosis*		
YPIII	S	[48]
*Escherichia coli*		
40309	R	A clinical isolate
*Staphylococcus aureus*		
Sau01	R	A clinical isolate
*Acinetobacter baumannii*		
Aba02	R	A clinical isolate

^a^ R, resistant; S, susceptible.

**Table 2:** Antimicrobial susceptibility tests of the *Klebsiella pneumoniae* strains used in this study.

Group	Antimicrobial Agent	Disk Content	Antibiotic Resistance ^a^			
			2106	0915	2404	1025
β-lactams	Aztreonam	30μg	R	I	R	R
	Piperacillin	100 μg	R	R	R	R
	Piperacillin-tazobactam	100/10 μg	R	R	R	R
	Cefazolin	30 μg	I	R	R	R
	Cefuroxime	30 μg	I	R	R	R
	Cefotaxime	30 μg	R	R	R	R
	Ceftriaxone	30 μg	I	R	R	R
	Cefepime	30 μg	I	I	R	R
	Cefoxitin	30 μg	S	R	R	R
	Cefoperazone-sulbactam	75/30 μg	S	I	I	R
	Ampicillin-sulbactam	10/10 μg	S	R	R	R
Quinolones	Ciprofloxacin	5 μg	S	S	S	R
	Levofloxacin	5 μg	S	S	S	R
	Norfloxacin	10 μg	S	S	R	R
Carbapenems	Imipenem	10 μg	I	R	R	R
	Meropenem	10 μg	S	I	R	R
Aminoglycosides	Amikacin	30 μg	S	S	R	R
	Gentamicin	10 μg	S	S	R	R
	Tobramycin	10 μg	S	S	R	R
Tetracyclines	Tetracycline	30 μg	S	S	S	S
Folate pathway antagonists	Trimethoprim	5 μg	S	S	R	S

^a^ R, resistant; S, susceptible

## Supplementary Materials

Supplementary materials can be found at https://www.mdpi.com/2076-2607/8/4/542/s1.
