# Supplementary material for: Isolation and Characterization of a Novel Phage for Controlling Multidrug-Resistant Klebsiella pneumoniae
Source: Microorganisms. 2020 Apr 9;8(4):542. doi: 10.3390/microorganisms8040542 (PMC7232175; doi:10.3390/microorganisms8040542)
Supplement: Supplementary file 1 [file microorganisms-08-00542-s001.pdf]

Type of the Paper: Article

Isolation and characterization of a novel phage for controlling multidrug-resistant *Klebsiella pneumoniae*

Qin Peng[1], Meng Fang[1], Xushan Liu[1], Chunling Zhang[1], Yue Liu[1], Yihui Yuan[2,*]

[1]Ministry of Education Key Laboratory for Ecology of Tropical Islands, College of Life Sciences, Hainan Normal University, Haikou 571158, P. R. China

[2]State Key Laboratory of Marine Resource Utilization in South China Sea, Hainan University, Haikou 570228, P. R. China.

*Correspondence: yuanyh@hainu.edu.cn

8 **Additional Table S1. Genome feature of the vB_KleS-HSE3 phage**

Gene accession	Direction	Nucleotide position		Length (bp)	Function	Best match	E-value	Similarity
		start	end					
*gp*1	+	247	618	372	hypothetical protein	*Klebsiella* phage vB_Kp3[ALJ98213.1]	7.00E-30	43%
*gp*2	+	825	1565	741	DNA methylase	*Klebsiella* phage vB_Kp3[ALJ98156.1]	3.00E-171	91.08%
gp3	-	1737	3047	1311	hypothetical protein	No hits		
*gp*4	+	1785	2516	732	DNA methylase	*Klebsiella* phage vB_Kp3[ALJ98158.1]	8.00E-172	92.12%
*gp*5	+	3171	3806	636	hypothetical protein	*Klebsiella* phage vB_Kp3[ALJ98162.1]	8.00E-70	49.14%
*gp*6	+	3716	4087	372	hypothetical protein	*Klebsiella* phage vB_Kp3[ALJ98163.1]	2.00E-59	77.35%
*gp*7	+	4084	4584	501	endopeptidase	*Klebsiella* phage vB_Kp3[ALJ98164.1]	2.00E-70	80%
*gp*8	+	4581	5078	498	lysin	*Klebsiella* phage vB_Kp3[ALJ98165.1]	2.00E-110	93%
*gp*9	-	5337	6035	699	hypothetical protein	*Klebsiella* phage vB_Kp3[ALJ98167.1]	4.00E-118	77%
*gp*10	-	6046	6282	237	hypothetical protein	*Klebsiella* phage vB_Kp3[ALJ98168.1]	5.00E-34	71%
*gp*11	-	6279	6644	366	hypothetical protein	*Klebsiella* phage vB_Kp3[ALJ98169.1]	5.00E-59	71.28%
*gp*12	-	6706	6927	222	hypothetical protein	*Klebsiella* phage vB_Kp3[ALJ98170.1]	3.00E-42	88%
*gp*13	-	6924	7292	369	hypothetical protein	*Klebsiella* phage vB_Kp3[ALJ98171.1]	4.00E-45	65.34%
*gp*14	+	7267	7605	339	hypothetical protein	No hits		
*gp*15	-	7598	8080	483	hypothetical protein	*Klebsiella* phage vB_Kp3[ALJ98173.1]	6.00E-46	51.48%
*gp*16	-	8270	8608	339	hypothetical protein	*Klebsiella* phage vB_Kp3[ALJ98175.1]	3.00E-64	81.60%
*gp*17	-	8730	9116	387	hypothetical protein	*Klebsiella* phage vB_Kp3[ALJ98178.1]	3.00E-35	54.45%
*gp*18	-	9196	9414	219	hypothetical protein	*Klebsiella* phage vB_Kp3[ALJ98179.1]	3.00E-38	83.33%
*gp*19	-	9425	11092	1668	DNA primase/helicase	*Klebsiella* phage vB_Kp3[ALJ98180.1]	0	86%
*gp*20	+	9701	10108	408	hypothetical protein	No hits		
*gp*21	+	10221	10628	408	hypothetical protein	No hits		
*gp*22	-	11501	11704	204	hypothetical protein	No hits		
*gp*23	+	11648	11974	327	terminase small subunit	*Klebsiella* phage vB_Kp3[ALJ98181.1]	1.00E-61	82%

*gp*24	+	12055	12345	291	hypothetical protein	*Klebsiella* phage vB_Kp3[ALJ98182.1]	1.00E-04	28%
*gp*25	+	12523	12762	240	hypothetical protein	No hits		
*gp*26	-	12735	12968	234	hypothetical protein	No hits		
*gp*27	+	12967	14358	1392	terminase large subunit	*Klebsiella* phage vB_Kp3[ALJ98185.1]	0	93.12%
*gp*28	+	14318	15958	1641	portal protein	*Klebsiella* phage vB_Kp3[ALJ98186.1]	0	82.72%
*gp*29	-	15071	15613	543	hypothetical protein	No hits		
*gp*30	+	15958	17076	1119	minor head protein	*Klebsiella* phage vB_Kp3[ALJ98187.1]	0	90%
*gp*31	-	17087	17695	609	hypothetical protein	No hits		
*gp*32	+	17108	17806	699	hypothetical protein	*Klebsiella* phage vB_Kp3[ALJ98188.1]	5.00E-136	86%
*gp*33	+	17899	19020	1122	major head protein	*Klebsiella* phage vB_Kp3[ALJ98189.1]	0	90.21%
*gp*34	+	19067	19525	459	hypothetical protein	*Klebsiella* phage vB_Kp3[ALJ98190.1]	4.00E-96	85.26%
*gp*35	+	19525	19908	384	hypothetical protein	*Klebsiella* phage vB_Kp3[ALJ98191.1]	4.00E-77	87%
*gp*36	+	19908	20591	684	hypothetical protein	*Klebsiella* phage vB_Kp3[ALJ98192.1]	1.00E-145	93%
*gp*37	+	20588	21040	453	hypothetical protein	*Klebsiella* phage vB_Kp3[ALJ98193.1]	9.00E-97	89%
*gp*38	+	21105	21716	612	major tail protein	*Klebsiella* phage vB_Kp3[ALJ98194.1]	8.00E-134	88%
*gp*39	+	21774	22163	390	hypothetical protein	*Klebsiella* phage vB_Kp3[ALJ98195.1]	7.00E-72	79.20%
*gp*40	+	22473	25319	2847	tail length tape measure protein	*Klebsiella* phage vB_Kp3[ALJ98197.1]	0.00E+00	93%
*gp*41	+	25231	25542	312	hypothetical protein	*Klebsiella* phage vB_Kp3[ALJ98198.1]	2.00E-15	34.40%
*gp*42	-	25502	25756	255	hypothetical protein	No hits		
*gp*43	+	25714	26985	1272	minor tail protein	*Klebsiella* phage vB_Kp3[ALJ98199.1]	0.00E+00	88%
*gp*44	-	26602	27111	510	hypothetical protein	No hits		
*gp*45	+	27443	28042	600	tail assembly protein	*Klebsiella* phage vB_Kp3[ALJ98200.1]	6.00E-128	85%
*gp*46	+	28027	28626	600	tail assembly protein	*Klebsiella* phage vB_Kp3[ALJ98201.1]	4.00E-133	94%
*gp*47	+	28626	32864	4239	tail fiber protein	*Klebsiella* phage vB_Kp3[ALJ98202.1]	0.00E+00	89.10%
*gp*48	+	32770	33720	951	tail fiber protein	*Klebsiella pneumoniae* ATCC	1.00E-178	89.18%

						43816[CEL82033.1]		
*gp*49	+	33617	38041	4425	tail fiber protein	*Klebsiella pneumoniae* ATCC 43816[CEL82033.1]	0	94.09%
*gp*50	-	35359	35898	540	hypothetical protein	*Acinetobacter baumannii*[CVI03216.1]	2.00E-05	23.40%
*gp*51	-	37544	38044	501	hypothetical protein	No hits		
*gp*52	+	38079	38417	339	tail fiber protein	*Klebsiella pneumoniae*[WP_064149004.1]	3.00E-58	85.14%
*gp*53	+	38451	39716	1266	tail fiber protein	*Klebsiella* phage vB_Kp3[ALJ98203.1]	0	88%
*gp*54	-	39743	40015	273	hypothetical protein	*Escherichia coli* [WP_023062971.1]	3.00E-04	25.50%
*gp*55	-	40012	40221	210	helix-turn-helix transcriptional repressor	*Klebsiella* phage vB_Kp3[ALJ98204.1]	3.00E-27	64.99%
*gp*56	-	40231	40710	480	single-stranded DNA-binding protein	*Klebsiella* phage vB_Kp3[ALJ98205.1]	3.00E-77	78%
*gp*57	-	40723	41412	690	recombinase	*Klebsiella* phage vB_Kp3[ALJ98206.1]	7.00E-152	90%
*gp*58	-	41469	42512	1044	exonuclease	*Klebsiella* phage vB_Kp3[ALJ98207.1]	0.00E+00	91%
*gp*59	-	42512	42874	363	hypothetical protein	*Klebsiella* phage vB_Kp3[ALJ98208.1]	2.00E-27	40.05%
*gp*60	+	42939	43247	309	hypothetical protein	No hits		
*gp*61	+	43136	43591	456	hypothetical protein	No hits		
*gp*62	-	43348	45396	2049	DNA helicase	*Klebsiella* phage vB_Kp3[ALJ98210.1]	0.00E+00	91%
*gp*63	+	44590	45162	573	hypothetical protein	No hits		
*gp*64	-	45384	45791	408	hypothetical protein	No hits		
*gp*65	+	45471	46037	567	hypothetical protein	*Klebsiella* phage vB_Kp3[ALJ98211.1]	6.00E-98	71.38%
*gp*66	+	46034	46621	588	DNA primase/helicase	*Klebsiella* phage vB_Kp3[ALJ98212.1]	3.00E-81	56.09%
*gp*67	-	46099	46695	597	methyltransferas	*Klebsiella pneumoniae* ISC21[CDL50211.1]	6.00E-60	47.04%

Table S2. The core genes of the *Klebsiella* phage vB_KleS-HSE3, vB_Kp3, ATCEA85, and 48ST307

Klebsiella phage vB_KleS-HSE3	*Klebsiella* phage vB_Kp3	*Klebsiella* phage ATCEA85	*Klebsiella* phage 48ST307
*gp*40	ALJ98197.1	QGJ86737.1	AQN32343.1
*gp*43	ALJ98199.1	QGJ86735.1	AQN32344.1
*gp*45	ALJ98200.1	QGJ86780.1	AQN32345.1
*gp*47	ALJ98202.1	QGJ86778.1	AQN32337.1
*gp*53	ALJ98203.1	QGJ86775.1	AQN32349.1

Table S3. The core genes of the *Klebsiella* phage vB_KleS-HSE3, vB_Kp3, and ATCEA85

Klebsiella phage vB_KleS-HSE3	*Klebsiella* phage vB_Kp3	*Klebsiella* phage ATCEA85
*gp*1	ALJ98213.1	QGJ86766.1
*gp*2	ALJ98156.1	QGJ86765.1
*gp*4	ALJ98158.1	QGJ86764.1
*gp*5	ALJ98162.1	QGJ86763.1
*gp*7	ALJ98164.1	QGJ86762.1
*gp*8	ALJ98165.1	QGJ86761.1
*gp*9	ALJ98167.1	QGJ86760.1
*gp*10	ALJ98168.1	QGJ86755.1
*gp*11	ALJ98169.1	QGJ86759.1
*gp*13	ALJ98171.1	QGJ86758.1
*gp*15	ALJ98173.1	QGJ86756.1
*gp*17	ALJ98178.1	QGJ86754.1
*gp*19	ALJ98180.1	QGJ86753.1
*gp*23	ALJ98181.1	QGJ86751.1

*gp*24	ALJ98182.1	QGJ86750.1
*gp*27	ALJ98185.1	QGJ86748.1
*gp*28	ALJ98186.1	QGJ86747.1
*gp*30	ALJ98187.1	QGJ86746.1
*gp*32	ALJ98188.1	QGJ86745.1
*gp*33	ALJ98189.1	QGJ86744.1
*gp*34	ALJ98190.1	QGJ86743.1
*gp*35	ALJ98191.1	QGJ86742.1
*gp*36	ALJ98192.1	QGJ86741.1
*gp*37	ALJ98193.1	QGJ86740.1
*gp*38	ALJ98194.1	QGJ86739.1
*gp*39	ALJ98195.1	QGJ86738.1
*gp*40	ALJ98197.1	QGJ86737.1
*gp*41	ALJ98198.1	QGJ86736.1
*gp*43	ALJ98199.1	QGJ86735.1
*gp*45	ALJ98200.1	QGJ86780.1
*gp*46	ALJ98201.1	QGJ86779.1
*gp*47	ALJ98202.1	QGJ86778.1
*gp*53	ALJ98203.1	QGJ86775.1
*gp*56	ALJ98205.1	QGJ86774.1
*gp*57	ALJ98206.1	QGJ86773.1
*gp*58	ALJ98207.1	QGJ86772.1
*gp*62	ALJ98210.1	QGJ86768.1
*gp*65	ALJ98211.1	QGJ86769.1
*gp*66	ALJ98212.1	QGJ86770.1

Table S4. The genomic information of the *Klebsiella* phage used for the dot plotting analysis.

Genome	Host	Accession	Length	Location	Classification	Isolation source
Klebsiella phage Spivey	*K. pneumoniae*	MK630230.1	110,659 bp linear DNA	USA	Siphoviridae; Sugarlandvirus	wastewater treatment plant
Klebsiella phage Sugarland	*K. pneumoniae*	MG459987.2	111,103 bp linear DNA	USA	Siphoviridae;Sugarlandvirus	activated sludge from a wastewater treatment plant
Klebsiella phage vB_Kpn_IME260	*K. pneumoniae*	NC_041899.1	123,490 bp linear DNA	China	Siphoviridae; Sugarlandvirus	-
Klebsiella phage KpGranit	*K. pneumoniae*	MN163280.1	122,710 bp linear DNA	Israel	Siphoviridae; Sugarlandvirus	-
Klebsiella phage KPN4	*K. oxytoca*	MN101228.1	108,916 bp linear DNA	Australia	Siphoviridae; Sugarlandvirus	-
Klebsiella phage vB_KpnS_FZ41	*K. pneumoniae*	MK521907.1	106,104 bp linear DNA	Russia	Siphoviridae; Sugarlandvirus	sewage water
Klebsiella phage AmPh_EK80	*K. pneumoniae*	MN434094.1	112,215 bp circular DNA	Australia	Siphoviridae; Sugarlandvirus	wastewater
Klebsiella phage JIPh_Kp127	*K. pneumoniae*	MN434096.1	113,671 bp circular DNA	Australia	Siphoviridae; Sugarlandvirus	wastewater
Klebsiella phage phiKO2	*K. oxytoca*	NC_005857.1	51,601 bp linear DNA	USA	Siphoviridae	-
Klebsiella phage 48ST307	*K. pneumoniae*	KY271402.1	52,338 bp linear DNA	Italy	Siphoviridae	-
Klebsiella phage ST846-OXA48phi9.1	*K. pneumoniae*	MK416021.1	38,370 bp linear DNA	Spain	Siphoviridae	-
Klebsiella phage 020009	*K. pneumoniae*	CP038007.1	55,032 bp linear DNA	China	Siphoviridae	-
Klebsiella phage ATCEA85	*K. aerogenes*	MN656993.1	47484 bp circular DNA	Korea	Siphoviridae	-
Klebsiella phage vB_KleS-HSE3	*K. pneumoniae*	MT075871	46747 bp linear DNA	China	Siphoviridae	wastewater
Klebsiella phage vB_Kp3	*K. pneumoniae*	KT367887.1	48,493 bp linear DNA	Switzerland	Siphoviridae	wastewater plant
Klebsiella phage KPN N54	*K. pneumoniae*	MF415413.1	59,100 bp circular DNA	Korea	Siphoviridae	-
Klebsiella phage Seifer	*K. pneumoniae*	MH817999.1	58,197 bp linear DNA	USA	Siphoviridae.	wastewater
Klebsiella phage YMC15/11/N53_KPN_BP	*K. pneumoniae*	MF476924.1	59,100 bp circular DNA	Korea	Siphoviridae	-
Klebsiella phage KPN U2874	*K. pneumoniae*	MF415411.1	59,087 bp circular DNA	Korea	Siphoviridae	-
Klebsiella phage KPN N137	*K. pneumoniae*	MF415410.1	59,100 bp circular DNA	Korea	Siphoviridae	-

Klebsiella phage KPN N98	*K. pneumoniae*	MG835858.1	59,214 bp linear DNA	Korea	Siphoviridae	-
Klebsiella phage Soft	*K. pneumoniae*	MN106244.1	57,805 bp linear DNA	USA	Siphoviridae	municipal wastewater
Klebsiella phage YMC16/01/N133_KPN_BP	*K. pneumoniae*	MF476925.1	58,387 bp linear DNA	Korea	Siphoviridae	-
Klebsiella phage ST899-OXA48phi17.2	*K. pneumoniae*	MK433583.1	17,998 bp linear DNA	Spain	Siphoviridae	-
Klebsiella phage ST101-KPC2phi6.3	*K. pneumoniae*	MK416017.1	43,942 bp linear DNA	Spain	Siphoviridae	-
Klebsiella phage ST147-VIM1phi7.2	*K. pneumoniae*	MK448232.1	34,200 bp linear DNA	Spain	Siphoviridae	-
Klebsiella phage 1 LV-2017	*K. pneumoniae*	KY271401.1	29,880 bp linear DNA	Italy	Siphoviridae	-
Klebsiella phage ST13-OXA48phi12.2	*K. pneumoniae*	MK422452.1	34,141 bp linear DNA	Spain	Siphoviridae	-
Klebsiella phage ST13-OXA48phi12.4	*K. pneumoniae*	MK422450.1	59,049 bp linear DNA	Spain	Siphoviridae	-
Klebsiella phage ST974-OXA48phi18.2	*K. pneumoniae*	MK448237.1	51,967 bp linear DNA	Spain	Siphoviridae	-
Klebsiella phage ST846-OXA48phi9.2	*K. pneumoniae*	MK416022.1	57,402 bp linear DNA	Spain	Siphoviridae	-
Klebsiella phage ST16-OXA48phi5.1	*K. pneumoniae*	MK416013.1	57,025 bp linear DNA	Spain	Siphoviridae	-
Klebsiella phage ST405-OXA48phi1.3	*K. pneumoniae*	MK416008.1	32,010 bp linear DNA	Spain	Siphoviridae	-
Klebsiella phage 5 LV-2017	*K. pneumoniae*	KY271399.1	47,014 bp linear DNA	Italy	Siphoviridae	-
Klebsiella phage ST16-OXA48phi5.3	*K. pneumoniae*	MK416014.1	29,301 bp linear DNA	Spain	Siphoviridae	-

Klebsiella phage KPP5665-2	*K. pneumoniae*	MF695815.1	39,241 bp linear DNA	Germany	Siphoviridae	-
Klebsiella phage ST11-VIM1phi8.2	*K. pneumoniae*	MK448234.1	48,230 bp linear DNA	Spain	Siphoviridae	-
Klebsiella phage ST13-OXA48phi12.5	*K. pneumoniae*	MK714353.1	44,913 bp linear DNA	Spain	Siphoviridae	-
Klebsiella phage 2b LV-2017	*K. pneumoniae*	KY271395.1	44,279 bp linear DNA	Italy	Siphoviridae	-
Klebsiella phage ST101-KPC2phi6.1	*K. pneumoniae*	MK448231.1	48,131 bp linear DNA	Spain	Siphoviridae	-
Klebsiella phage ST11-VIM1phi8.1	*K. pneumoniae*	MK448233.1	42,666 bp linear DNA	Spain	Siphoviridae	-
Klebsiella phage 2 LV-2017	*K. pneumoniae*	KY271396.1	44,400 bp linear DNA	Italy	Siphoviridae	-
Klebsiella phage ST512-KPC3phi13.1	*K. pneumoniae*	MK448235.1	42,666 bp linear DNA	Spain	Siphoviridae	-
Klebsiella phage KLPN1	*K. pneumoniae*	NC_028760.1	49,037 bp linear DNA	Ireland	Siphoviridae; Tunavirinae	cecal effluent
Klebsiella phage KP36	*K. pneumoniae*	NC_029099.1	49,797 bp linear DNA	Poland	Siphoviridae; Tunavirinae; Webervirus	-
Klebsiella phage Sanco	*K. pneumoniae*	MK618657.1	48,790 bp linear DNA	USA	Siphoviridae; Tunavirinae; Webervirus	wastewater treatment plant
Klebsiella phage Sin4	*K. pneumoniae*	MK931442.1	49,916 bp linear DNA	USA	Siphoviridae; Tunavirinae; Webervirus	waste water treatment plant- influent
Klebsiella phage Skenny	*K. pneumoniae*	MK931444.1	49,935 bp linear DNA	USA	Siphoviridae; Tunavirinae; Webervirus	waste water treatment plant- activated sludge
Klebsiella phage Sweeny	*K. pneumoniae*	MK931443.1	50,241 bp linear DNA	USA	Siphoviridae; Tunavirinae; Webervirus	waste water treatment plant- influent
Klebsiella phage Shelby	*K. pneumoniae*	MK931445.1	49,045 bp linear DNA	USA	Siphoviridae; Tunavirinae; Webervirus	pond water
Klebsiella phage KOX1	*K. oxytoca*	KY780482.1	50,526 bp circular DNA	Australia	Siphoviridae; Tunavirinae; Webervirus	wastewater sample

Klebsiella phage Sushi	*K. pneumoniae*	NC_028774.1	48,754 bp linear DNA	USA	Siphoviridae; Webervirus	Tunavirinae;	-
Klebsiella phage MezzoGao	*K. pneumoniae*	MF612072.1	49,807 bp linear DNA	USA	Siphoviridae; Webervirus	Tunavirinae;	-
Klebsiella phage 1513	*K. pneumoniae*	NC_028786.1	49,462 bp linear DNA	China	Siphoviridae; Webervirus	Tunavirinae;	-
Klebsiella phage 13	*K. pneumoniae*	MK170446.1	43,094 bp linear DNA	Hungary	Siphoviridae; Kp36virus	Tunavirinae;	-
Klebsiella phage GH-K3	*K. pneumoniae*	MH844531.1	49,427 bp linear DNA	China	Siphoviridae; Webervirus	Tunavirinae;	sewage
Klebsiella phage NJS1	*K. pneumoniae*	MH445453.1	49,292 bp linear DNA	China	Siphoviridae; Webervirus	Tunavirinae;	domestic sewage
Klebsiella phage JY917	*K. pneumoniae*	MG894052.1	37,655 bp linear DNA	China	Siphoviridae; Webervirus	Tunavirinae;	-
Klebsiella phage KPN N141	*K. pneumoniae*	MF415412.1	49,090 bp circular DNA	Korea	Siphoviridae; Webervirus	Tunavirinae;	-
Klebsiella phage NJS3	*K. pneumoniae*	MH633486.1	49,387 bp linear DNA	China	Siphoviridae; Webervirus	Tunavirinae;	hospital sewage
Klebsiella phage TAH8	*K. pneumoniae*	MH633484.1	49,344 bp linear DNA	China	Siphoviridae; Webervirus	Tunavirinae;	hospital sewage
Klebsiella phage KpKT21phi1	*K. pneumoniae*	MK278861.1	49,106 bp circular DNA	Israel	Siphoviridae; Webervirus	Tunavirinae;	-
Klebsiella phage NJS2	*K. pneumoniae*	MH633485.1	50,132 bp linear DNA	China	Siphoviridae; Webervirus	Tunavirinae;	sewage water
Klebsiella phage TSK1	*K. pneumoniae*	MH688453.1	49,861 bp linear DNA	Pakistan	Siphoviridae; Webervirus	Tunavirinae;	-

Klebsiella phage PKP126	*K. pneumoniae*	NC_031053.1	50,934 bp linear DNA	Korea	Siphoviridae; Webervirus	Tunavirinae;	-
Klebsiella phage vB_KpnS_SegesCirculi	*K. pneumoniae*	MN013080.1	50,713 bp linear DNA	USA	Siphoviridae; Webervirus	Tunavirinae;	raw sewage
Klebsiella phage vB_KpnS_Call	*K. pneumoniae*	MN013079.1	51,487 bp linear DNA	USA	Siphoviridae; Webervirus	Tunavirinae;	raw sewage
Klebsiella phage vB_KpnS_IMGroot	*K. pneumoniae*	MN013076.1	52,866 bp linear DNA	USA	Siphoviridae; Webervirus	Tunavirinae;	raw sewage
Klebsiella phage KL	*K. pneumoniae*	MN379832.1	47,844 bp circular DNA	USA	Siphoviridae; Webervirus	Tunavirinae;	-
Klebsiella phage vB_KpnS_Penguinator	*K. pneumoniae*	MN013087.1	51,678 bp linear DNA	USA	Siphoviridae; Webervirus	Tunavirinae;	raw sewage
Klebsiella phage vB_KpnS_Alina	*K. pneumoniae*	MN013083.1	51,780 bp linear DNA	USA	Siphoviridae; Webervirus	Tunavirinae;	sewage
Klebsiella phage vB_KpnS_KingDDD	*K. pneumoniae*	MN013078.1	51,562 bp linear DNA	USA	Siphoviridae; Webervirus	Tunavirinae;	raw sewage
Klebsiella phage vB_KpnS_Domnhall	*K. pneumoniae*	MN013075.1	54,438 bp linear DNA	USA	Siphoviridae; Webervirus	Tunavirinae;	raw sewage
Klebsiella phage KOX9	*K. oxytoca*	MN101222.1	52,904 bp linear DNA	Australia	Siphoviridae; Eclunavirus	Tunavirinae;	-
Klebsiella phage vB_KpnS_KpV522	*K. pneumoniae*	KX237515.1	51,099 bp linear DNA	Russia	Siphoviridae; Webervirus	Tunavirinae;	sewage
Klebsiella phage vB_KpnS_FZ10	*K. pneumoniae*	MK521904.1	50,381 bp linear DNA	Russia	Siphoviridae; Webervirus	Tunavirinae;	sewage water
Klebsiella phage SH-Kp 160016	*K. pneumoniae*	KY575286.1	49,170 bp circular DNA	China	Siphoviridae; Webervirus	Tunavirinae;	sewage water

Klebsiella virus GML-KpCol1	*K. pneumoniae*	MG552615.1	50,249 bp linear DNA	Turkey	Siphoviridae; Tunavirinae; Webervirus	Kayseri municipal wastewater treatment plant
Klebsiella phage ST13-OXA48phi12.3	*K. pneumoniae*	MK422451.1	84,199 bp linear DNA	Spain	Siphoviridae; Nickievirus	-
Klebsiella phage YX3973	*K. pneumoniae*	MK327140.1	46,907 bp linear DNA	China	Siphoviridae; Roufvirus	medical sewage
